# Vibration Cycling Did Not Affect Energy Demands Compared to Normal Cycling During Maximal Graded Test

**DOI:** 10.3389/fphys.2019.01083

**Published:** 2019-08-23

**Authors:** Monèm Jemni, Yaodong Gu, Qiuli Hu, Michel Marina, Mohamed Saifeddin Fessi, Wassim Moalla, Bessem Mkaouer, Ferman Konukman

**Affiliations:** ^1^Faculty of Sports Science, Ningbo University, Zhejiang, China; ^2^Institut Nacional d’Educació Física de Catalunya, Barcelona, Spain; ^3^UR EM2S, High Institute of Sport and Physical Education Sfax, University of Sfax, Sfax, Tunisia; ^4^Higher Institute of Sport and Physical Education of Ksar Saïd, Manouba University, Manouba, Tunisia; ^5^Sport Science Program, College of Arts and Science, Qatar University, Doha, Qatar

**Keywords:** cycloergometer, VO_2*max*_, ventilatory threshold, OBLA, energy demands

## Abstract

The aim of this study was to compare the physiological responses between a vibration induced cycling step protocol (Vib) and normal cycling (without vibration, no-Vib). Eighteen moderate trained males (age 24.1 ± 4.3 years; weight 76.5 ± 10.5 kg; height 178.0 ± 6.4 cm) have participated in this study. They randomly performed two gradual maximal exercise tests on two separate days using a new bike that automatically induces vibration cycling and the Corival cycle ergometer. The choice of two different bikes was made because of the impossibility to recreate the same power output without altering the cycling cadence on the vibration Bike. Both protocols were matched for power output and cycling cadence incrementations. Oxygen uptake (VO_2_), carbon dioxide production (VCO_2_), ventilation (VE), heart rate (HR), blood lactate and rating of perceived exertion (RPE) during each stage were continuously recorded. No statistical differences were founded for all variables when comparing the Vib to no-Vib trials, except a higher ventilation during the vibration trial at submaximal levels. The results of this study do not confirm those of previous studies stated that Vib increased metabolic demands during cycling exercise. Added vibration stimulus to an incremental cycling protocol does not affect physiological parameters.

## Introduction

Exercise and activity in today’s society incorporate a wide variety of training types, techniques and equipment. New training methods are constantly being introduced and revised, targeting numerous different facets of the exercise spectrum, from improving fitness levels and performance capabilities in elite athletes to injury prevention and medical therapy. One of the most interesting and potentially important current topics amongst sport scientists, physiotherapists and coaches is vibration (Vib) exercise (Cardinale and Bosco, 2003). A particular interest has been given to vibration cycling by different clinicians, medicals and fitness practitioners (Sperlich et al., 2009; Filingeri et al., 2012; Munera et al., 2017). The use of whole-body and/or localized vibrations as means for enhancing athletic performance is a recent development in exercise physiology (Sands et al., 2006; Sperlich et al., 2009; Filingeri et al., 2012; Munera et al., 2017), being only commercially available since the start of the millennium. Several gymnasiums are now equipped with vibration platforms and/or portable devices used for localized vibration to the latests bikes that integrate vibration within the cycling gears. Traditionally, vibration is an oscillation determined by peak-to-peak movement, frequency, amplitude, and maximum acceleration. It has been suggested that incorporating Vib into exercise evokes greater muscle contraction than the same exercise performed without Vib. This mechanical stimulus could be applied to the whole body, or to some parts of the body (Sands et al., 2006; Eckhardt et al., 2011). It is understood that mechanical vibration applied directly to the muscle can elicit a reflex muscle contraction named “Tonic Vibration Reflex” also known as “Tonic Vibration Stretch Reflex” (Hagbarth and Eklund, 1966; Cochrane, 2011). Tonic Vibration Reflex in turn, activates a large number of motoneurons and leads to the recruitment of previously inactive muscle fibers. These late have indeed suggested a possible neural mechanism responsible of the effect induced by whole-body vibration. It is very important to investigate the effects of any novel training regime on the physiological variables of the human beings. This will enable its validation and to save the trainees and their coaches’ time. One of the main objectives of any training is to give the body a *‘unambiguous message’* enabling quick and smooth specific adaptations, and hence better performance. This study is indeed focuses on the effects of vibration cycling on different physiological variables of the cardiorespiratory and the metabolic systems.

Numerous studies have indeed concluded that Vib training has various physiological benefits, including increased bone mineral density, strength, cardio respiratory fitness, increased blood flow and hormonal responses (Verschueren et al., 2004; Gusi et al., 2006; Iodice et al., 2010; Hunter et al., 2011); these findings have contributed to consider and support the use of Vib as a new training stimuli (Sands et al., 2006; Suhr et al., 2007; Rittweger, 2010). The integration of vibration into cycling has been inspired not only by the road cyclists but also from the mountain bikers. Paris-Roubaix, for example, is a road cycling 1-day race that contains a significant part with pebbles. Cyclists feel the vibrations and they are always intrigued by any similar specific training that could prepare them for this race without been on the track.

Nevertheless, the few authors (Samuelson et al., 1989; Mester et al., 2006; Suhr et al., 2007; Sperlich et al., 2009) who used a vibration cycle ergometer to assess physiological responses, concluded that incorporating a Vib stimulus into dynamic cycling exercise, such as riding on a cycle ergometer, elicits greater physiological responses than cycling without Vib. Sperlich et al. (2009) showed an increased in the VO_2__*max*_ performing a maximal incremental protocol on a bike where the frame was mounted on a Vib platform, compared to normal cycling. However, Samuelson et al. (1989) showed that Vib reduces the work capacity during an incremental cycling exercise to exhaustion. Cycling time was indeed reduced by 13 min when vertical vibration stimulus was applied through the pedals. This finding could lead to a significant reduction in the exercise duration while having the same benefits of normal exercise. This could also lead to apply vibration exercise mode to those who cannot exercise for a long time, either for medical reasons or for time constraint.

Previously, Filingeri et al. (2012) demonstrated that adding Vib to incremental cycling tests seems to elicit a quicker energetic demand during the maximal graded exercise test. However, it seems like a crucial methodological error has slipped away from the authors’ sight. While trying to maintain the same pedaling rate and the same resistance gear on the Powerbike the authors did not realize that the power output was completely different because of the vibration induced through the pedals. Our team have worked on the same powerbike and tried to reproduce the same power outputs, but these were not possible without simultaneously altering the cycling cadence and the resistance gears in both conditions. The only solution to recreated comparable cycling conditions was by choosing a different bike, in our case, we have chosen the Lode Corival cycle ergometer. The recent paper published by Munera et al. (2017) have even triggered more curiosity to dig-in little further into the Filingeri et al.’s (2012) article; Munera et al. (2017) compared several vibration frequencies (20, 30, 40, 50, 60, and 70 Hz) while pedaling at a constant power output (150 W) and a pedaling cadence of 80 RPM during 6-min with a similar non-vibration condition. The authors showed that there was no significant influence of vibrations on heart rate (HR) and oxygen uptake (VO_2_) by adding vibration to cycling. However, muscular activity has significantly increased with the vibration stimulus [even though the increase was very low (<1%)]. The authors have only exposed their participants to a short period of vibration (6 min) and in separate bouts, hence the question that remains is would there be any difference if exposed to longer periods and at higher pedaling and cycling powers? We thought we would add a significant understanding of the entire effect of the vibration stimulus if participants are exposed to increasing intensities rather than cycling at constant speed. Therefore, the aim of the present study was to monitor the physiological responses (cardiorespiratory and metabolic) and the perceived effort related to adding Vib to cycling exercise compared to a standard cycle ergometer during a maximal incremental exercise test with similar power outputs and pedaling frequencies. We hypothesized that vibrating cycling exercise will induce the same effects, i.e., increases oxygen consumption, blood lactate production, and perceived effort, compared to non-vibrating cycling exercise. This hypothesis is based on the physiological reasoning that vibration would induce more neuromuscular unit recruitment compared to non-vibration.

## Materials and Methods

### Participants

Eighteen healthy and moderately trained male subjects (6–8 h of training per week) volunteered to participate in this study (age 24.1 ± 4.3 years; weight 76.5 ± 10.5 kg; height 178.0 ± 6.4 cm). Each subject signed a consent form after reading an information sheet and being verbally informed by the principal investigator. The experimental protocol was performed in accordance with the Declaration of Helsinki for human experimentation and was approved by the ethical committee of the University of Greenwich.

### Study Design

Each participant randomly performed two maximal graded cycling tests (with and without Vib). Tests were performed at the same time of the day with a recovery of 72 h. Participants were invited to keep the same food habits during the intervention and were instructed to have their last meal 2 h minimum before testing. They have also been asked to refrain from any vigorous physical activity in the 24 h before testing sessions. The cardiorespiratory response, metabolic response and perceived exertion were assessed throughout the tests.

#### Pre-testing

several pre-testing procedures have taken place including the assessment of the real power output developed by the *PowerBIKE^TM1^*, but also the short-term reliabilities of the bikes by performing the same experimentation 48 h later. Note only nine participants have participated in this process. The cycling tests were the same as described below but the subjects were not pushed to their maximal exhaustion as the objectives were to standardaise the procedure and to minimize technical errors.

### Maximal Graded Cycling Tests With and Without Vibration

**FIGURE 1:**
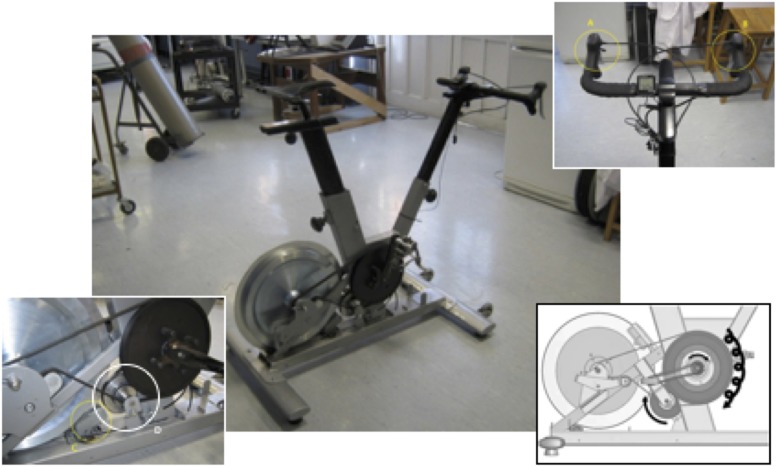
The *powerBIKE*^TM^ (early design). **(A–C)** The handlebar manual commands and the magnet responsible for the resistance system and gear. **(D)** The vibration mechanism (crank).

Each participant performed two maximal graded cycling tests (with and without Vib). The vibrating cycle ergometer used was a *powerBIKE*^TM^ prototype (Figure 1) (Power Plate, Netherlands), whilst a Lode Corival cycle ergometer (REF. 20103498, Netherlands) was used for the no vibration trial. Power output and cadence were matched in both conditions through meticulous pre-trials that required the intervention of the original manufacturer’s engineers. Gear 4 was chosen as a set load all the way through the protocol, as it is an average vibration stimulus, not too easy and not too challenging for those who are not confirmed cyclists. The bike generates vibrations from the gearbox; it could be calibrated to the range of (*f* = 25 to 45 Hz) and peak-to-peak displacement from 2 to 10 mm, depending on the chosen setting (resistance pedaling gear and cycling cadence). For the purpose of this current study, a frequency (*f*) and peak-to-peak displacement of 23.3–33.3 Hz and 2 mm, respectively were applied. These variables were accurately measured during the protocol using accelerometers and high frequency video shootings. They are indeed complying with Hagbarth and Eklund (1966) and Zaidell et al. (2013) recommendations to induce the greatest neuromuscular activity, although this later studied the vibration applied directly to the muscle and reported a greater muscle activation during loaded leg in Anterior Tibialis and SOL at 50 Hz than 25 HZ. Vibration was transmitted from the bike’s crank to the pedals and then to the lower limbs. Note that the pedals were normal ones; no cycling shoes with cleats were allowed in order to ensure similar conditions with the other bike. Vibration could be activated and/or deactivated with a small command near the breaks located at bike’s handle. The crank (axis) is located in an eccentric housing. When the Vib is not activated the housing is still and the crank and pedals rotate in a normal circular path (Figure 1). However, when the Vib system is activated, the clutch engages a second belt that is connected to the housing and starts to rotate at 20 times per revolution. This is indeed the mechanism that causes the crank and pedals to move off the rotation center and to vibrate while pedaling. In a simpler way: it is the extra rotations applied to the rotating crank that create the vibration. As the pedals are attached to the crank, they would vibrate too. Rotating vibration goes all directions and is orientated to vibrate the lower body muscle groups. The manufacturer has ensured that vibration would not affect much of the other body parts by inserting “vib absorbers” in the frame.

**FIGURE 2:**
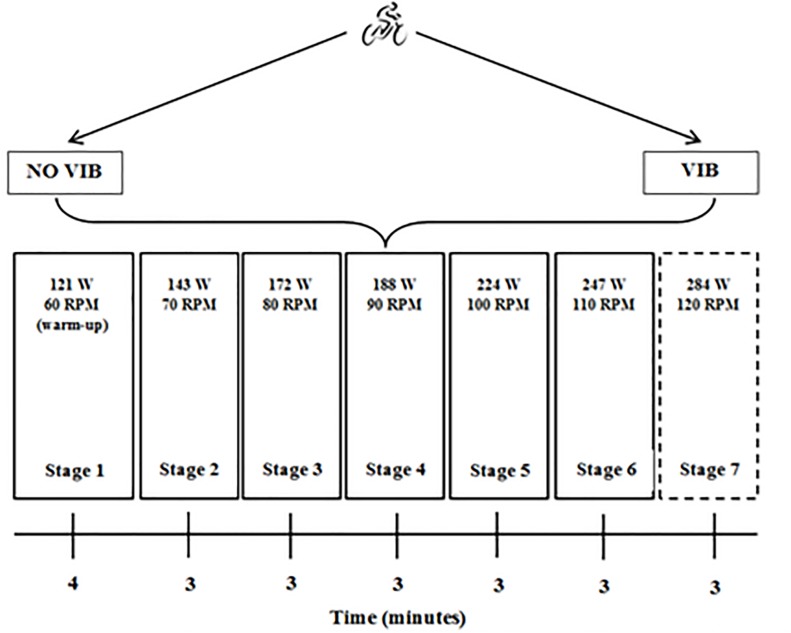
Graded maximal cycling test protocols to assess VO_2_max with or without vibration.

Cycling cadence was directly measured on the bike. The protocol was a progressive and maximal, started with 4 min warm up at 60 rev.min^–1^ at gear 4 of the *powerBIKE*^TM^ (comparable to 121 watts of work); subsequent increases were 10 rev.min^–1^ (rpm) every 3 min until exhaustion (Figure 2). Measuring the power output on the PowerBike was only made possible by referring to the manufacturer’s engineers. Each stage’s power output and cycling cadences were matched between both conditions.

The settings of the two bikes (*powerBIKE*^TM^ and *Lode*)^TM^ were adjusted according to the participants’ physical measurements, including the height of the seat, the distance between the handlebar and the seat, seat to center of the crank, handlebar to the center of the crank and the handlebar to the floor. The measurement process was identical at each trial.

### Cardiorespiratory Responses

Heart rate (HR) was continuously assessed with a HR belt transmitter fitted around the upper chest (RS300X, Polar, Finland). Oxygen uptake (VO_2_), carbon dioxide production (VCO_2_), ventilation (VE) and the rate of the expiratory ratio (RER) were continuously assessed with an online gas analyzer Vacumed Metabolic Measurement System (Metamax, Cortex, Germany) monitored by a TurboFit software, V. 5.0 (United States). Respiratory variables were normalized to the basal condition and expressed in percentage (NVO_2__*max*_, NVCO_2__*max*_, NVE_*max*_, NHR_*max*_, NL_*max*_, and RER_*max*_) and also expressed relative to body mass (VO_2__*bm*_ and VCO_2__*bm*_ in ml.kg^–1^.min^–1^); and VE_*bm*_ in l.kg^–1^.min^–1^).

### Perceived Exertion

Rating of perceived exertion (RPE) was collected during the last 30 s of each stage of the maximal graded cycling exercise and also at the end of the test (RPE max) using the 6–20 Borg Scale (Borg, 1970).

### Metabolic Responses

Blood lactate concentration (mmol.l*^–^*^1^) was assessed in 5 μl of blood samples taken from the fingertip at the end of each stage using lactate analyzers (Biosen EKF diagnostic, Germany). Two metabolic thresholds were then assessed: the onset of blood lactate accumulation (OBLA) and the ventilatory threshold (VT). OBLA was determined according to Sjödin and Jacobs (1981) and VT was determined visually by checking the breakpoints of the curve VE/VO_2_ (Posner et al., 1987). The correspondent percentages of VO_2_max and HRmax, as well as Blood lactate and RPE, were then calculated.

### Statistical Analysis

Data are reported as a mean ± standard deviation and confidence intervals were set at 95% level (95% CI) using the statistical software SPSS Statistics for Windows, version 20 for Windows® (SPSS Inc., Chicago, IL, United States). The normality of distribution, verified with the Shapiro-Wilk test, was acceptable for all raw variables except RPE and RER. The non-parametric condition persisted in these two variables even after successive statistical transformations. The *t*-test was used to compare differences between the Vib and no-Vib trial in the raw parametric variables (VO_2_, VCO_2_, HR, BL, VE, VT, and OBLA), whereas the Wilcoxon Rank-sum test was applied in the raw non-parametric variables (RPE and RER). The normal distribution was confirmed with all normalized variables with respect to the basal condition. Two kinds of regression analysis were carried out to calculate the relationship (best fit method) between the workload and the metabolic variables: (1) On one side, a linear regression was used for the VO_2_, the VCO_2_, the HR and the RPE; (2) On the other side, an exponential model (*y* = 3.2831⋅e^0.0041*x*^) was chosen to characterize the relationship between the intensity and the VE and the blood lactate. The average slope of the group was then calculated to compare the trials. For normalization of the slope, the cross-multiplication method was used. Absolute and relative reliability and variability were assessed using the typical error of measurement (TEM), the coefficient of variation (CV) and the intra-class correlation coefficient (ICC) (Hopkins, 2000). Effect size (Es) was also calculated to estimate the power of the analysis based on the sample size. The following scale was used to interpret ES: [(small: 0.1–0.33), (moderate: 0.34–1.00)]. Statistical significance was accepted at *P* ≤ 0.05.

## Results

### Pre-tests

Several subjects have participated in the pre-trials to determine the reliability of the data on each bike. Table 1 shows the overall statistical analysis when comparing the same pre-test repeated twice either on the PowerBike^TM^ or on the Corival ergometer. The TEM, the CV, the ICC are all in acceptable range and the effect size is moderate.

**TABLE 1:** Reliability assessment during the pre-trials.

**Pre-Trial 1 vs. Pre-Trial 2**	**Wilcoxon rank-sum test (*P*)**	**TEM**	**TEM%**	**CV (%)**	**ICC**	**Effect size (Es)**
PowerBike	0.062	55.39	0.74	2.08	0.99	0.34
Corival ergometer	0.062	40.06	0.43	2.83	0.99	0.34

### Cardiorespiratory Response

**FIGURE 3:**
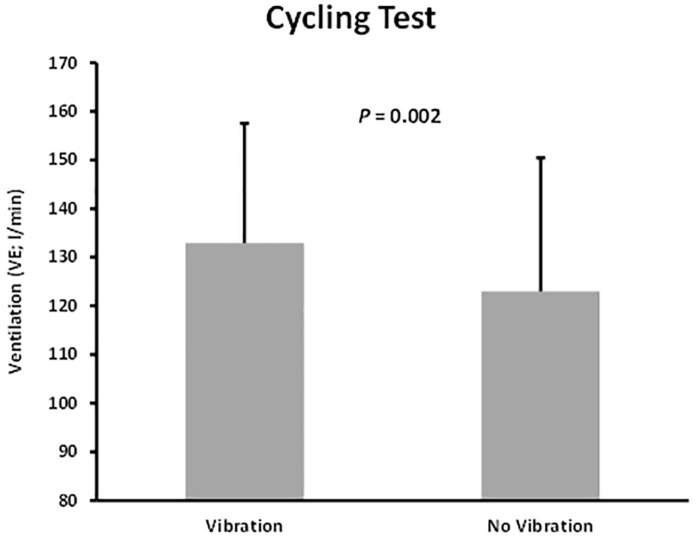
Differences of ventilation between the vibration and no vibration conditions during graded maximal cycling test.

The analysis did not show any significant differences between the two trials (Vib and no-Vib) when comparing the raw data of the variables except for the VE (Table 2) which had higher values during the Vib trial (Figure 3).

**TABLE 2:** Paired samples *t*-test comparison between vibration and no vibration conditions.

**Variables**	**Mean difference**	**Confidence interval of the difference**	***t***	***P***	***Es***
VO_2_max	1.31	−0.23–2.85	1.79	0.091	0.76
VCO_2_max	1.81	−0.20–3.82	1.90	0.074	0.76
VE_*max*_	9.95	4.05–15.85	3.56	0.002	0.56
HR_*max*_	−0.39	−4.31–3.53	−0.21	0.837	0.87
L_*max*_	0.71	−0.98–2.40	0.89	0.386	0.89
RER_*max*_	0.01	−0.02–0.04	0.54^Ψ^	0.599	0.75
RPE_*max*_	−0.50	−1.25–0.25	−1.41^Ψ^	0.177	0.88

*The variables are the maximal value achieved during or after the execution of the test. Variables are maximal relative oxygen consumption (VO_2_max), maximal relative CO_2_ consumption (VCO_2_max), maximal ventilation (VE_*max*_), maximal heart rate (HR_*max*_), maximal venous blood lactate concentration (L_*max*_), maximal rate expiration ratio (RER; CO_2_/O_2_), and rating of perceived exertion (RPE), ^Ψ^ Wilcoxon ran sum.*

**FIGURE 4:**
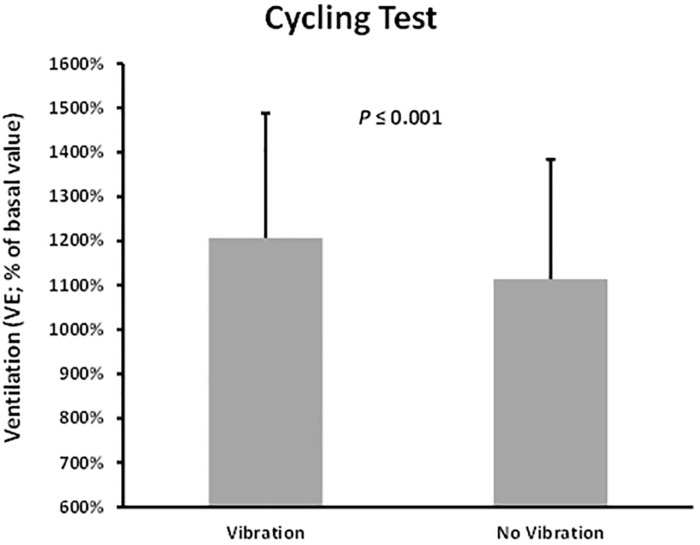
Differences of relative ventilation with respect to basal value (%) between the vibration and no vibration conditions during graded maximal cycling test.

Comparison of the normalized variables between the trials using their absolute values confirmed that the unique significant difference between both trials was the VE (Table 3 and Figure 4). Moreover, when respiratory variables were normalized and reported to body mass (VO_2__*bm*_, VCO_2__*bm*_ and VE_*bm*_ respectively), the VE was again the only variable showing a significant difference (*P* = 0.003) between Vib and no-Vib trials. For interest, there were no significant differences neither in VO_2__*bm*_ (*P* = 0.122) nor in VCO_2__*bm*_ (*P* = 0.67) between the trials.

**TABLE 3:** Paired samples *t*-test comparison between vibration and no vibration conditions.

**Variables**	**Mean difference**	**Confidence interval of the difference**	***t***	***P***	***Es***
NVO_2max_	15.4%	−8.2–38.9%	1.38	0.185	0.75
NVCO_2max_	25.6%	−6.0–57.1%	1.71	0.106	0.75
NVE_*max*_	94.3%	42.1–146.4%	3.82	0.001	0.56
NHR_*max*_	−0.8%	−6.2–4.7%	−0.30	0.767	0.88
NL_*max*_	34.5%	−177.8–246.8%	0.34	0.736	0.89
RER_*max*_	0.7%	−1.9–3.4	0.56^Ψ^	0.580	0.77

*The variables are normalized with respect to basal condition (%). Normalized variables are maximal relative oxygen consumption (NVO_2_max), maximal relative CO_2_ consumption (NVCO_2_max), maximal ventilation (NVE_*max*_), maximal heart rate (NHR_*max*_), maximal venous blood lactate concentration (NL_*max*_), and maximal rate expiration ratio (NRER; CO_2_/O_2_), ^Ψ^ Wilcoxon ran sum.*

**FIGURE 5:**
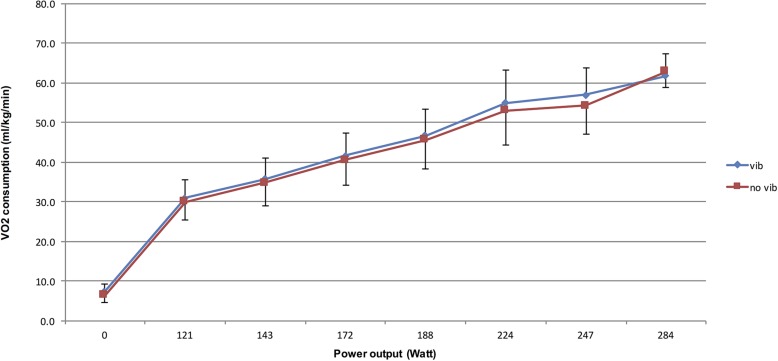
Kinematics of the oxygen consumptions during the vibration and no vibration conditions during graded maximal cycling test.

**FIGURE 6:**
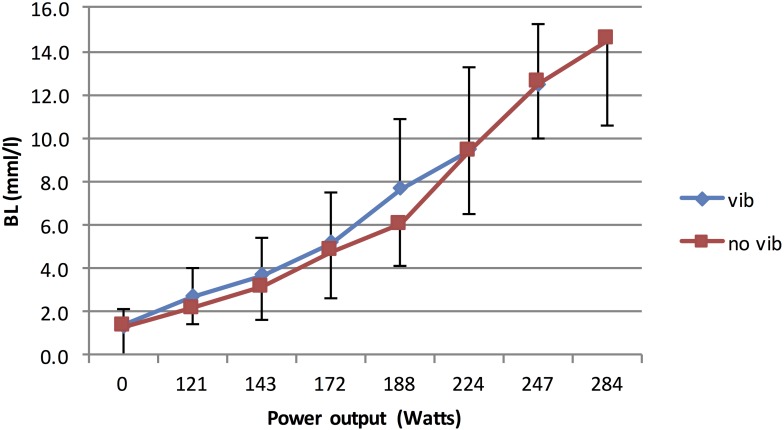
Blood lactate during the vibration and no vibration conditions during graded maximal cycling test.

Figure 5 shows the kinematics of the oxygen consumptions whereas Figure 6 shows the blood lactate evolution during both trials.

The individual slope coefficients were also calculated as the value of the “best fit” regression model obtained from each individual. No significant differences were observed when comparing the linear models (Table 4). In fact, vib trial did not induce higher slope of VO_2_, VCO_2_, HR, RER, and RPE. Exponential models have been used to calculate the VE and the blood lactate slopes. Comparison between the trials showed higher VE values during the Vib trial, but no difference between blood lactate concentrations (Table 4).

**TABLE 4:** Paired samples *t*-test comparison between vibration and no vibration conditions.

**Slope coefficients**	**Mean difference**	**Confidence interval of the difference**	***t***	***P***	***Es***
† SVO_2_	0.002	−0.009–0.012	0.352	0.729	0.76
† SVCO_2_	0.005	−0.006–0.0155	0.911	0.375	0.76
◆ SVE	0.046	0.009–0.071	2.854	0.032	0.56
† SHR	−0.001	−0.032–0.030	−0.056	0.956	0.87
◆ SBL	−0.021	−0.043–0.008	−1.764	0.312	0.89
† RER	0.001	−0.002–0.001	0.408^Ψ^	0.689	0.75
† SRPE	−0.003	−0.013–0.007	−0.713^Ψ^	0.486	0.88

*Slope coefficient (a) that characterize the equation’s linearity (*y* = ax + b) or the exponential regression (*y* = 3.2831e^0.0041*x*^) of each variable during the incremental cycling test. Parameters are the slope coefficient from the linear regression (†) or exponential regression (◆) calculated for each variable. Relative oxygen consumption (SVO_2_), relative CO_2_ consumption (SVCO_2_), ventilation (SVE), heart rate (SHR), blood lactate concentration (BL), rate expiration ratio (RER; CO_2_/O_2_), and rating of perceived exertion (SRPE), ^Ψ^ Wilcoxon rank sum.*

### Metabolic Response

The OBLA was achieved at 57.9 ± 1.5% and 55.4 ± 0.22% of the VO_2_ max; whereas the VT was achieved at 53.7 ± 2.6% and 58.5 ± 1.2% of the VO_2_ max respectively for the Vib and the noVib trials. All relevant variables calculated at both thresholds are shown in Table 5. There were no significant differences when comparing these variables between the trials, neither at the OBLA nor at the VT.

**TABLE 5:** OBLA and VT variables during the vibration and no vibration trials.

	**OBLA**		**VT**	
		
	**Vib**	**No Vib**	**Vib**	**No Vib**
Power (W)	172	172	168 ± 6	172 ± 1
Cadence (rpm)	80	80	70 ± 6	80 ± 2
Time (min)	9	10	8 ± 2	9 ± 1
VO_2_ (ml.kg^–1^.min^–1^)	37.7 ± 2.2	38.1 ± 1.3	33.2 ± 4	36.8 ± 2
%VO_2max_	57.9	55.4	53.7	58.5
HR (bpm)	151.5 ± 1.4	152.4 ± 1.5	137.4 ± 6.8	144.6 ± 2.2
RPE (Borg scale)	14.5 ± 2.5	14.3 ± 2	12.5 ± 3.4	14.3 ± 2

## Discussion

The aim of the present study was to monitor the physiological responses (cardiorespiratory and metabolic) and the perceived effort related to adding Vib to cycling exercise using a modified stationary bike. We were expecting some real differences between the condition, however, the main outcome showed that vibration-cycling physiological responses did not differ to normal cycling, with the exception of a significantly higher VE. Therefore, the null hypothesis is accepted.

The outcome of a single acute bout of vibration cycling exercise, when compared with no-Vib, did not produce significant variation in VO_2__*max*_ values. These results are in line with other investigations reporting that a single acute session of exercise does not represent a sufficient stimulus to significantly increase the maximal aerobic capacity (Garber et al., 2011). It is evident that significant training quality and quantity are required to improve the physiological components of VO_2__*max*_, i.e., functional capacity and the cardiovascular system. Moreover, HRmax did not significantly differ between the Vib and noVib trial, confirming that subjects had experienced similar cardiovascular stress during both protocols. Note that all subjects have cycled until exhaustion as confirmed by the RPE_*max*_ values recorded at the end of each trial (Lagally et al., 2002). The fact that blood lactate values obtained at the end of both trials were not significantly different, assumes that subjects achieved similar muscular acidosis in both protocols. These results are different to the ones presented by Filingeri et al. (2012) (conducted on the same *powerBIKE*)^TM^ who reported higher values of RPE, blood lactate, HR and VO_2_ consumption during the vibration cycling test. Our investigation showed that the only variable that presented a significant difference between the two trials was the ventilation VE, [either in absolute value (l.min^–1^), or relative to basal value at rest (%) or even when reported to body mass (l.min^–1^⋅kg^–1^)]. No other significant differences were found following analysis of the cardiorespiratory peak values (VO_2__*max*_, VCO_2__max_, HR_max_), metabolic (BL_max_, OBLA and VT) and perceived exertion (RPE).

Vib exposure implicated more or less the same aerobic and anaerobic demands on the body. This result could be explained by firstly, the lack of significant differences between the trials’ VO_2__*max*_, VCO_2__*max*_, HRmax, L_*max*_ and RPE values; and secondly, the lack of significant differences in time, cadence and stage at which OBLA and VT were reached. Although the p values of the VO_2_ max and VCO_2_ max are border line for significance (respectively 0.091 and 0.074), we cannot deny the normalized data in Table 3 and mainly the slope comparison in Table 4 that have both shown far larger p values. This thorough analysis confirms that the VO_2_ and the VCO_2_ have indeed very similar patterns from the start and until the very end of the tests in both conditions. On the other hand, we do all know how difficult is to achieve a proper VO_2_ max in particular for non-experiences endurance athletes. Although, most of the tested subjects have indeed reached their VO_2_ max criteria, we do very much relay on the submaximal data, which are less debatable. These findings disagree with Filingeri et al. (2012) and might be explained by the fact that the application of Vib as an external stimulus does not produce significantly greater or quicker energy demands to the body and thus does not contribute to a perception of the greater workload after an acute bout. Therefore, it can be suggested that the additional “workload” incurred with Vib does not affect the cardiovascular and pulmonary systems, as confirmed by the non-significant difference in VO_2__*max*_ and blood lactate values between the Vib and no Vib trials. The extrapolation of these findings could indeed contrast the theory that vibration exposure increases the fast twitch neuromuscular unit recruitments (mainly type IIa and IIb fibers; Hagbarth and Eklund, 1966), known to have a role in high-intensity muscle contraction (Fitts and Widrick, 1996). However, this extrapolation cannot be confirmed until investigating the neuromuscular variables in a perfect match condition. Such muscle fibers operate predominantly during high-intensity effort via the glycolytic pathway, in which lactic acid production often exceeds clearance and leads to higher lactate accumulation (Kohn et al., 2011). This physiological state is thought to slow the glycolytic process and may be a limiting factor to performance (Lagally et al., 2002). However, this physiological mechanism was not more evident during our Vib trial when compared to the non-vibration. Moreover, the acidosis generated during both Vib and no-Vib trial might not be the only cause of skeletal muscle fatigue. The Vib stimulus might have generated and influenced muscle fatigue in other ways.

Samuelson et al. (1989) demonstrated when exercising at a constant submaximal workload, Vib may negatively affect cycling performance in terms of exercise duration. The same authors have suggested that the mechanism responsible for this effect may not be related to central physiological adaptations, but could be localized within the working muscles. Furthermore, the present study also contrasts with Sperlich et al. (2009) results that showed a significant increase in VO_2__*max*_ at higher workloads of 250 and 300 W during cycling with vibration compared to normal cycling. Nevertheless, Sperlich et al.’s (2009) protocol used a different Vib frequency (*f*) and peak-to-peak displacement (20 Hz and 4 mm) compared to the present study (23.3 to 33.3 Hz and 2 mm respectively).

Different vibration frequencies, different peak to peak time and also different exposure times to vibration can significantly affect the physiological responses; therefore, the differences in the Vib settings used in our study compared to Sperlich et al.’s (2009) could be the reason for the contracting physiological responses. Furthermore, few subjects have managed to cycle at higher powers (stage 6 and 7; 247 W and 284 W) in the present study seeing the reduction in the working time, as confirmed by previous studies (Samuelson et al., 1989; Filingeri et al., 2012). This point was not clarified in Sperlich et al. (2009) study, in addition to the fact that their bike was held on a vibration platform, hence vib frequency and peak-to-peak displacement could be absorbed by the bike’s frame before being transmitted to the subject. The vibration generated by the PowerBike^TM^ is self-generated and mainly orientated to vibrate the lower body muscle groups. The manufacturer has ensured that vibration would not affect much of the other body parts by inserting “vib absorbers” in the frame.

We do however acknowledge some limitations of this present study. The inability to match the power outputs between both conditions whilst preserving the same cycling cadence on the Powerbike^TM^ has obliged the research team to compare these physiological markers between the PowerBike^TM^ and the Lode Corival cycle ergometer. In reality, we have tried many different bikes but for reliability and reproducibility issues we have chosen the Corival ergometer as it one of the global standard bikes that is available in most of the exercise physiology labs. Nevertheless, we made the impossible to match as many variables as we could between both of them. As mentioned above, we went up to recall the conceptual engineers to calculate the power output generated by the PowerBike^TM^ at each of the set gears versus different pedaling frequencies. This has enabled us to match the power output and the frequencies between both bikes. In fact, the matched setting is considered the novelty in the investigation compared to previous studies.

Furthermore, the results of the present study could have been affected by the cycling technique difference between the two ergometers. In fact, the *powerBIKE*^TM^ is designed for maintaining an ergonomic posture similar to the cyclist technique whereas, the Corival is designed for a general population that needs physical exercise aiming for different purposes (such as medical reason or improving fitness level); therefore, cycling ergonomics could be different, although subjects were encouraged to adopt almost the same position on either bike to ensure a fair comparison. In addition, none can deny the effects of various psycho-physiological factors (such as motivation, quality of sleep, etc…) that we could not totally control during the 72 h between the two test conditions on the results. These points could indeed be considered as a limitation of this current study.

The protocol applied in this study could be considered as the first attempt at an intervention into vibration cycling using this specific device (*powerBIKE*)^TM^, thus further studies should be conducted to examine the effects of applying a vibratory stimulus to other forms of dynamic exercise to allow evaluation of additional effects in human body such as strength, bone mineral density, angiogenic factors, blood flow, cardiorespiratory fitness and other benchmarks for human performance. In addition, few studies that incorporate vibration within dynamic submaximal exercise for longer durations (T 30 > min) are very scarce; so, the effect of vibration cycling on prolonged exercise is still unknown. Because of this limitation, as well as those stated above, there is a clear warrant for further research into the use of the *powerBIKE*^TM^ and the relationship between Vib and physiological responses.

## Conclusion

The main purpose of this study was to compare the effect of vibration induced cycling on cardiorespiratory and metabolic variables during a maximal incremental exercise versus a normal cycling test matched for power and cadence. The experimental hypothesis of the present study suggested that vibration protocol performed on the *powerBIKE*^TM^ would significantly increase the physiological variables compared to normal cycling due to a greater neuro-muscular recruitment. Unfortunately, the results of this study do not confirm this hypothesis. Although this study highlighted a slightly higher ventilation during the Vib trial submaximal stages, a thorough analysis of the data showed that adding a vibration stimulus to cycling did not significantly affect the cardiovascular and the metabolic responses compared to normal cycling. Subjects’ VO_2__*max*_ was not significantly altered by the Vib stimulus (around 62.3 ml.kg^–1^.min^–1^ in both trials). Similarly, metabolic thresholds (OBLA and VT) were achieved at almost identical levels (57.9 and 55.4% for OBLA; 53.7 and 58.5% for VT) respectively for the vibration and non-vibration trials. According to the above, we are not sure that vibration cycling would replace normal cycling to achieve a quicker benefit. Its application in real world should be done with caution unless to reduce the exercise time compared to normal cycling, something that professional athletes could find interesting in particular during the competitive season where time is scarce because of the travel schedule. Nonetheless, further investigations should deepen the various physiological and neuromuscular incurred.

## Data Availability

All datasets generated for this study are included in the manuscript and/or the supplementary files.

## Ethics Statement

Each subject signed a consent form after reading an information sheet and being verbally informed by the principal investigator. The experimental protocol was performed in accordance with the Declaration of Helsinki for human experimentation and was approved by the ethical committee of the University of Greenwich.

## Author Contributions

All authors contributed considerably to the manuscript and approved the final submission. MJ lead author and director of the investigation series, conceived and designed the study, collected and analyzed the data, and reviewed and edited the manuscript. YG, QH, MM, WM, BM, and FK analyzed the data, and reviewed and edited the manuscript. MF analyzed the data, reviewed and edited the manuscript, and managed the manuscript submission.

## Conflict of Interest Statement

MJ is the Director of the International Science and Football Academy (Ltd.). This company had no role and no relation to the study (neither in the design, data collection and analysis, decision to publish, or preparation of the manuscript). The remaining authors declare that the research was conducted in the absence of any commercial or financial relationships that could be construed as a potential conflict of interest.

## Footnotes

1 All necessary permissions were obtained from the manufacturer to use the brand name.
